# Premature Ovarian Insufficiency in a Patient With Vogt–Koyanagi–Harada Syndrome: An Unusual Association—A Case Report

**DOI:** 10.1155/carm/2727070

**Published:** 2026-07-16

**Authors:** Ruan Barboza Rocha, Denise Corrêa Benzaquem, Paloma de Sousa Passos, Evellyn Karine Cruz da Silva, Jadiele Barbosa Mendonça, Paula Rita Leite da Silva, Jorge Frank Braga Ferreira, Cleiton Fantin

**Affiliations:** ^1^ Laboratory of Cytogenetics and Cytogenomics, State University of Amazonas, Manaus, Brazil, ufam.edu.br; ^2^ Department of Gynecology, Getúlio Vargas University Hospital, Manaus, Brazil; ^3^ Central Animal Facility, Federal University of Amazonas, Manaus, Brazil, ufam.edu.br

**Keywords:** medical diagnosis, premature ovarian insufficiency, rare diseases, Vogt–Koyanagi–Harada

## Abstract

Premature ovarian insufficiency (POI) is a clinical syndrome characterized by ovarian failure in women of reproductive age before the age of 40 years. Its main manifestations include menstrual cycle disturbances, such as oligomenorrhea or amenorrhea. The etiology of POI is highly heterogeneous, with a particular emphasis on genetic and immunological factors. In addition, the condition may be associated with other rare diseases, such as Vogt–Koyanagi–Harada (VKH) syndrome. Our study aimed to describe a rare clinical case of a patient from Manaus, Amazonas, Brazil, who was simultaneously diagnosed with two rare conditions: POI and VKH syndrome, with a specific focus on their etiological characteristics, clinical manifestations, and laboratory findings. Clinical data were collected from the patient’s medical record, as registered in the outpatient clinic database at Araújo Lima Hospital. Case description: The case involves a 38‐year‐old, nulliparous, mixed‐race, married female patient diagnosed with POI and VKH syndrome. The understanding of these rare syndromes and the search for an integrated approach are fundamental to ensure better clinical outcomes, quality of life, and reproductive planning for affected patients. This report provides relevant information on the association between two rare syndromes, with the aim of contributing to medical decision‐making and diagnostic clarification, as well as providing guidance on appropriate multidisciplinary follow‐up. Thus, early diagnosis and appropriate treatment are essential steps in promoting this patient’s health.

## 1. Introduction

Premature ovarian insufficiency (POI) is a clinical syndrome defined by the loss of ovarian function in women of childbearing age before the age of 40. The main symptoms of POI are menstrual disorders such as amenorrhea or oligomenorrhea, in addition to elevated levels of gonadotropins and low levels of estradiol. These symptoms result in a hypoestrogenic and hypergonadotropic clinical picture [1, 2]. The first reports of this syndrome date back to 1942 and were made by Fuller Albright, who named the condition “primary ovarian insufficiency” [3].

POI is to be differentiated from natural‐age menopause, as women with POI have unique needs and management options. They may not only suffer from symptoms associated with estrogen deficiency but may also experience additional health issues with a significant impact on their quality of life and later health outcomes. POI affects fertility, bone health, cardiovascular health, sexual function, psychological health, and neurological function, making it a challenge for patients and healthcare professionals (HCPs) [4, 5].

Approximately 1% of women before the age of 40% and 0.1% of women before the age of 30 are affected by POI [6]. Furthermore, according to epidemiological data, the occurrence of POI may be associated with ethnicity [7]. A cross‐disciplinary epidemiological survey was conducted to determine eligibility for participation in a prospective, multiethnic, and multidisciplinary study funded by the Study of Women’s Health Across the Nation (SWAN). This study identified the prevalence of POI as 1% among Caucasian women, 1.4% among African Americans, 1.4% among Hispanics, 0.5% among Chinese women, and 0.1% among Japanese women [8].

Among the most important etiological causes are genetic factors; numerical and structural chromosomal abnormalities involving the X chromosome; and metabolic, infectious, and iatrogenic factors [9]. Most of the causes of POI remain undefined; however, it is estimated that between 4% and 30% of the cases have an autoimmune origin [1, 10].

In 1906, Alfred Vogt in Switzerland first described a patient with premature whitening of eyelashes of sudden onset and bilateral subacute iridocyclitis. Twenty years later, Harada (1926) reported a case series with bilateral serous retinal detachment in association with cerebrospinal fluid (CSF) pleocytosis. Shortly thereafter (1929), Koyanagi published a review article associating unequivocally the posterior eye involvement with auditory and integumentary manifestations. In 1932, Babel suggested that these cases represented a single entity, which was then named Vogt–Koyanagi–Harada (VKH) disease [11, 12].

VKH syndrome is a rare, autoimmune, multisystemic disorder that primarily targets melanocyte‐rich tissues, including retina, meninges, skin, and inner ear [12]. The disease is often characterized by a systemic inflammatory response that leads to a range of clinical manifestations, including anterior uveitis, auditory disturbances (such as tinnitus and hypoacusis), alopecia, and skin depigmentation [13].

VKH progresses through distinct stages: the prodromal stage marked by systemic viral‐like symptoms; the acute stage which is characterize by bilateral chorioretinitis and panuveitis; the chronic stage that includes pigmentation changes such as vitiligo and the characteristic “sunset glow” fundus; and the recurrent stage, defined by repeating episodes of anterior uveitis and chronic complications like choroidal neovascularization [13, 14].

Most studies have found that women were affected more frequently than men and that most patients were in the second to fifth decades of life at the onset of the disease [15]. However, children and the elderly may also be affected [15, 16]. Its etiological cause is linked to immunological factors that act in the autoimmune process, mediated by T cells directed against melanocytes [17]. It is believed that damage to the melanocyte surface is caused by cytotoxicity mediated by CD4+ Th1 lymphocytes in individuals with genetic predisposition, in particular, the presence of the HLA‐DRB1∗0405 allele [16–18].

We report the case of a 38‐year‐old woman from Manaus, Amazonas, Brazil, who was simultaneously diagnosed with two rare conditions: POI and VKH syndrome. This report highlights the etiological aspects as well as the main clinical and laboratory findings. Given the scarcity of similar descriptions in the literature, this case contributes to a better characterization and improved clinical management of these two conditions.

## 2. Case Presentation

A 38‐year‐old nulliparous female patient of mixed race, married, born and residing in Manaus, Amazonas, was evaluated. Clinical data were obtained through a review of the patient’s medical records available in the database of the Araújo Lima Outpatient Clinic at the Hospital Universitário Getúlio Vargas (HUGV). No written consent has been obtained from the patient as there are no patient identifiable data included in this case report. Imaging studies were not available for review to complement the clinical analysis. At the age of 30 years, she presented to the Araújo Lima Outpatient Clinic and was evaluated in the climacteric unit by the Gynecology and Breast Health team, with complaints of secondary amenorrhea, mastalgia, persistent hot flashes, irritability, chronic pelvic pain, and infertility.

After the evaluation of the medical history and physical examination, hypotrophy of the genitals was found, and, despite adequate hair removal, the vaginal walls and cervix were also observed to be hypotrophied, in addition to ovarian atrophy. On physical examination, the patient presented a fibroelastic consistency and decreased vaginal volume, with no other alterations.

Laboratory tests revealed slightly elevated glucose levels (115 mg/dL) and normal liver and kidney function, as well as cholesterol and triglyceride levels within normal limits. The complete blood count indicated mild leukopenia and the absence of significant alterations in the urine. Elevated levels of follicle‐stimulating hormone (111.8 mIU/mL) and low levels of estradiol (20 pg/mL) were also detected. At the age of 30, the patient was diagnosed with POI. The patient also had a family history of the condition, as her mother was diagnosed with POI at the age of 35.

At approximately 34 years of age, the patient developed an acute ophthalmologic condition characterized by progressive visual blurring, which evolved over a 10‐day period to significant loss of visual acuity. The clinical presentation was associated with blurred vision, severe headache, cervical pain, low back pain, and fever. Retinal detachment was initially suspected; however, following specialized evaluation at the HUGV, a diagnosis of VKH syndrome was established.

Currently, the patient is on continuous use of azathioprine, pregabalin, duloxetine, calcium carbonate, vitamin D, eye drops, and hormone replacement therapy. She reported gastrointestinal intolerance to amitriptyline and fluoxetine, and the use of both medications was discontinued.

**FIGURE 1:**
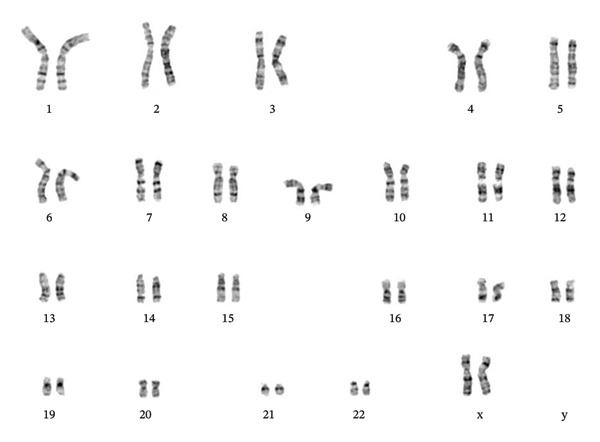
Conventional karyotyping using GTG banding showing a normal female chromosomal complement (46, XX), with no numerical or structural abnormalities.

The patient underwent cytogenetic analysis with a peripheral blood lymphocyte culture according to the methodology described by Moorhead et al. [19] and GTG banding for investigation of genetic etiology at the Laboratory of Human Cytogenetics of the Amazonas State University (UEA). The analysis of 50 metaphases revealed a karyotype of 46, XX in all analyzed cells, indicating the absence of numerical or structural chromosomal alterations, according to the methodology described by Seabright [20] (Figure 1).

## 3. Discussion

This report presents the case of a patient affected by two rare syndromes: POI and VKH syndrome, highlighting the importance of an accurate diagnosis in patients with these conditions. The management of these syndromes requires an interdisciplinary approach, involving areas such as gynecology, ophthalmology, dermatology, rheumatology, and neurology, according to the affected organs, with the aim of improving the prognosis and preventing sequelae that affect the physical and mental health of the patient.

Mukhtar and Gandhi [21] reported the association of these two conditions in a 28‐year‐old woman. The patient was diagnosed with VKH syndrome at the age of 14 years; subsequently, at 20 years of age, she was diagnosed with Graves’ disease and, at 25 years, she received a diagnosis of POI. This case highlights a rare presentation of VKH, Graves’ disease, and POI, suggesting that VKH may be part of a broader autoimmune polyglandular spectrum.

VKH syndrome is more common among Asians, Native Americans, Hispanics, Indians, and individuals from the Middle East; it is distinctly uncommon in White populations. The peak incidence occurs during the third decade of life, with a female predominance reported among North American patients [22]. A historical cross‐sectional study including 244 pediatric patients with noninfectious uveitis demonstrated that female sex, bilateral ocular involvement, anterior location, and a chronic clinical course were common features. The frequency of patients with idiopathic uveitis was slightly higher than that of patients with diffuse (nonidiopathic) uveitis [23].

POI, formerly known as premature ovarian insufficiency or premature menopause, is defined as the loss of ovarian function before the age of 40. The symptoms of POI are similar to those of the menopausal transition at a normal age. The short‐term consequences mainly result from the deficiency of estrogen. The main clinical manifestations are disorders of the menstrual cycle, such as primary or secondary amenorrhea. Other manifestations are also reported: vasomotor symptoms such as hot flushes, night sweats, insomnia, heart palpitations, headaches, vaginal dryness, dyspareunia, psychological distress, and sexual dysfunction [9, 24].

POI is a complex clinical syndrome with physiological and psychological consequences of multifactorial origin that affect the quality of life of young women of reproductive age. Many women often have feelings of depression, especially related to impaired fertility. For these women, infertility can be a significant problem although some of them may ovulate sporadically [25].

In addition, the consequences for women’s long‐term health include increased cardiovascular disease, increased risk of coronary and ischemic disease, decreased bone mineral density, increased risk of osteoporosis, progressive decline in fertility that may progress to primary or secondary infertility, vulvovaginal atrophy, neurological effects, and an overall reduction in life expectancy. Understanding and clarifying these risk factors facilitate the creation of prevention and risk minimization strategies to optimize the health of women with this condition [26–28].

To date, the etiology of most cases diagnosed as POI remains unclear. It is known that autoimmune diseases coexist in approximately 10%–55% of the patients with POI. Autoimmune disorders are assumed to trigger 4%–30% of the POI cases [29, 30]. The human ovary is commonly the target of an autoimmune attack that leads to ovarian dysfunction, which can manifest as POI, unexplained infertility, or endometriosis. The development of POI that is associated with autoimmune reactions is caused by alterations in T‐cell subsets and T‐cell–mediated injury, increased autoantibody‐producing B cells, a low number of suppressor/cytotoxic effector lymphocytes, and a decrease in the number and activity of natural killer cells [31].

Cytogenetic studies in patients affected by POI have shown a high prevalence of chromosomal abnormalities in both those with primary and those with secondary amenorrhea [32]. Investigations involving karyotype are justified in all cases associated with noniatrogenic POI, regardless of the age of the woman [2, 4]. G‐banded karyotyping should be requested since genetic and chromosomal causes correspond to an important portion of the etiology [6].

However, even in the face of normal karyotype test results, the possibility of genetic alteration cannot be ruled out since mutations in different genes may cause POI. The most common single‐gene cause that results in this condition is the premutation of the FMR1 gene, which is located on the long arm of chromosome X. This mutation is based on an expanded CGG trinucleotide repeat (55–199 repeats) in the untranslated region. Patients with this premutation have an increased risk of developing POI [7, 33]. Therefore, a more detailed investigation is necessary for this patient, involving tests using molecular techniques for diagnostic confirmation.

Complications of VKH syndrome that lead to visual loss include cataracts in about 25% of the patients, glaucoma in 33%, and subretinal neovascular membranes in about 10%; the latter, however, are an important cause of late visual loss [34]. Treatment should be started early and intensively, and rapid diagnosis is essential for the prognosis of the disease. The basis of treatment consists of the use of corticosteroids and immunosuppressants. Initially, systemic use in high doses is recommended, followed by oral administration. After improvement of the symptoms, discontinuation of treatment should be gradual, occurring over a period of 3–6 months, and treatment can be maintained for up to 1 year in cases of relapse [10]. The examinations used are optical coherence tomography, fluorescein angiography, and B‐scan ultrasound [35].

Clinically, VKH disease is divided into two phases: acute with first onset and chronic with repeated episodes [36]. The intensive use of combined steroidal and nonsteroidal immunosuppression for prolonged periods, initiated at the very beginning of treatment, is the usual regimen for VKH disease [37].

It has been shown that, in a small percentage of patients with VKH, late tapering of systemic corticosteroid therapy may lead to exacerbation since nonsteroidal immunosuppression is not related to this type of treatment, even when severe immunosuppressive therapy is started from the very beginning. On the other hand, rapid reduction is necessary when corticosteroid treatment is interrupted due to the onset of adverse effects such as gastrointestinal ulcers, diabetes, osteoporosis, and, less frequently, corticosteroid‐induced psychosis. The disease progresses to the chronic recurrent stage if adequate levels of inflammatory control and/or clinical quiescence are not achieved [38, 39].

The patient in our study presents a complex clinical picture, with multiple comorbidities, including autoimmune diseases and conditions associated with menopause, which require continuous multidisciplinary monitoring. She developed osteopenia and osteoarthritis in the lumbar spine (L5–S1) as a consequence of POI, which also resulted in infertility. In addition, she presented glaucoma and fibromyalgia as secondary complications of VKH syndrome, as well as a breast lump classified as BI‐RADS Category 3. The early diagnosis of these two rare diseases is essential in order to preserve the physical and mental health of patients, offering an etiological explanation and assisting in the prevention of bone and cardiovascular complications, as well as providing guidance on fertility options.

## 4. Conclusion

Finally, the diagnosis and treatment of POI and VKH syndrome require a multidisciplinary approach by specialists, aiming to provide comprehensive and personalized care to patients. The understanding of these rare syndromes and the search for an integrated approach are essential in order to ensure the best clinical outcome, quality of life, and reproductive planning for patients affected by these conditions.

This clinical case reports a 38‐year‐old patient diagnosed with POI at the age of 30, as well as VKH syndrome. The patient presented a complex clinical picture, with secondary amenorrhea, infertility, ovarian atrophy, and progressive ocular symptoms. In addition, she developed secondary complications related to POI, such as osteopenia and osteoarthritis, and complications related to VKH, such as glaucoma and fibromyalgia. Genetic investigation revealed a 46, XX karyotype, with no detectable chromosomal abnormalities. However, the possibility of genetic mutations associated with POI cannot be ruled out since alterations in different genes may be involved in the development of the condition.

## Funding

This study was funded by Fundação de Amparo à Pesquisa do Estado do Amazonas and Coordenação de Aperfeiçoamento de Pessoal de Nível Superior.

## Conflicts of Interest

The authors declare no conflicts of interest.

## Data Availability

Data sharing is not applicable to this article as no datasets were generated or analyzed during the current study.
